# Preparation and characterization of renal cell peptides from fetal rats for their antitumor activity

**DOI:** 10.1002/2211-5463.70075

**Published:** 2025-06-26

**Authors:** Zhe Zhang, Yuan Cao, Jing Du, Ying Zhang, Junxia Wang, Ying Yuan, Lianqing Sun

**Affiliations:** ^1^ Department of Traditional Chinese Medicine The First Affiliated Hospital of Medical School of Xi'an Jiaotong University Shaanxi China; ^2^ China Aviation Industry Corporation Xi'an Institute of Aeronautical Computing Technology Shaanxi China; ^3^ Department of Traditional Chinese Medicine East District of the First Affiliated Hospital of Medical School of Xi'an Jiaotong University Shaanxi China

**Keywords:** anti‐tumor effect, biologically active, fetal rats renal cell, peptides

## Abstract

Bioactive peptides with potent antitumor activity are attractive therapeutic agents. The present study aimed to prepare renal cell peptides (RCPs) from fetal rats to test their antitumor activities *in vitro*. Candidate peptides were characterized by capillary HPLC and MS and their bioactivity was predicted using PeptideRanker. The predicted top 10 bioactive peptides were synthesized and tested for their cytotoxicity against different types of tumor cells by cell counting kit‐8 assays and their half maximal inhibitory concentration values were calculated. Protease‐digested < 3 kDa protein products reduced the viability of Michigan Cancer Foundation (MCF)‐7 cells in a dose‐dependent manner. Functionally, many candidate peptides were predicted to have antitumor activity and the top ten peptides (RCPs 1–10) were synthesized. Interestingly, RCP1, 5 and 6 displayed preferable cytotoxicity against human cancer MCF‐7, A549, HCT‐116, Hela, HepG2 and SGC‐7901 cells and their cytotoxicity was dose‐dependent. RCPs prepared from fetal rats displayed potent cytotoxicity preferably against different types of cancer cells *in vitro* in a dose‐dependent manner which may be valuable for the treatment of malignant tumors.

## Graphical abstract

This study aimed to prepare renal cells (RCs) from fetal rats which were digested by enzymes. Candidate peptides RCPs were characterized by capillary HPLC and MS and their bioactivity was predicted using peptideranker. The predicted top 10 bioactive peptides were synthesized. Interestingly, RCP1, 5 and 6 displayed preferable cytotoxicity against different types of cancer cells *in vitro* in a dose‐dependent manner.

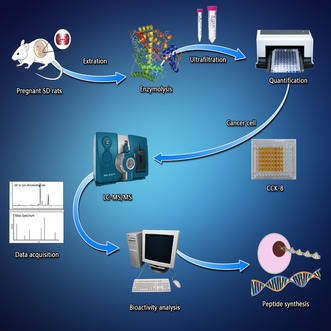

**Abbreviations**
5‐FU: 5‐fluorouracil
CCK‐8: cell counting kit‐8
IC_50_
: half maximal inhibitory concentration
MCF: Michigan Cancer Foundation
RCP: renal cell peptide
RC: renal cell
SD: Sprague–Dawley
THLE‐2: transformed human liver epithelial‐2

The therapeutic strategies for the treatment of solid malignant tumors mainly include surgery, radiotherapy, chemotherapy, endocrine therapy, targeted therapy and immunotherapy, among others. The traditional anti‐tumor chemotherapeutic drugs usually have characteristics of severe adverse effects and promoting drug resistance in the tumors. Hence, new therapeutic drugs with high efficacy and low adverse effects are urgently needed for the management of patients with malignant tumors in the clinic.

Biologically active peptides have been demonstrated to have certain physiological functions or to benefit the life activities of biological organisms [1]. These peptides can be naturally present or derived from animals, plants and microorganisms. These small molecular anti‐tumor peptides have attracted wide attention because of their unique characteristics, including their strong penetration ability, low toxicity, high efficiency and easy absorption by cancer cells. Compared with traditional chemotherapeutic drugs, bioactive peptides have a high preference against tumors, a high safety profile for tumor patients and can combine with chemotherapeutic or immunotherapeutic drugs because they can increase the sensitivity of tumors to chemotherapies and immunotherapies. Preclinical research and some clinical trials have shown that many bioactive peptides can inhibit tumor growth and metastasis through various pathways [2]. Bioactive peptides have gained significant attention in recent years for their potential as antitumor therapies. Indeed, bioactive peptides have been extracted from various organisms and artificially synthesized, and some of them have been applied in clinical practice. Carrera *et al*. [3] obtained two bioactive peptides that were homologous to the N‐terminus of anti‐tumor proteins by artificial synthesis and found that treatment with those bioactive peptides inhibited the growth of human cervical cancer Hela cells *in vitro* by 70% and the angiogenesis of human cervical tumors in mice. Komatsu *et al*. [4] synthesized a series of derivatives of cyclic peptide CHAP and found that CHAP31 was the most active and stable peptide. Treatment with CHAP31 significantly inhibited the growth of murine melanoma B16/BL6 tumors in nude mice. Laakkonen *et al*. [5] obtained a nine‐mer peptide LyP‐1 from the peptide library using phage display technology and found that intravenously injected peptide accumulated in the tumor microenvironment and induced the death of breast cancer cells in mice. However, new bioactive peptides are needed for the control of tumor growth.

Our previous studies have shown that extracts of renal cells (RCs) from fetal rats have a series of pharmacological activities, such as anti‐aging properties, immunoregulation, elevating serum sex hormone levels, improving osteoporosis, delaying renal failure and correcting anemia in rodents. Subsequently, we found that an RC peptide (RCP) from fetal rats inhibited the proliferation, invasion and migration of breast cancer Michigan Cancer Foundation (MCF)‐7 cells. However, the specific peptide components and detailed anti‐tumor activity are still unclear. The present study explored the efficacy of enzymatical extraction of RCPs from fetal Sprague–Dawley (SD) rats and tested their anti‐tumor activities in different types of tumor cells. Our data indicated that some RCPs had strong antitumor activities against the proliferation of human malignant tumor cells *in vitro* in a manner preferably against human cancer cells.

## Materials and methods

### Cell lines

Human breast cancer MCF‐7 (RRID:CVCL_0031), human lung cancer A549 (RRID:CVCL_0023), human colon cancer HCT‐116 (RRID:CVCL_0291), human cervical cancer Hela (RRID:CVCL_0030), human hepatoma HepG2 (RRID:CVCL_0027), human gastric cancer SGC‐7901 (RRID:CVCL_0520) and human non‐tumor mamma gland epithelial MCF‐10A (RRID:CVCL_0598) and transformed human liver epithelial‐2 (THLE‐2) (RRID:CVCL_3803) cells were obtained from Shanghai Donghuan Biotechnology (Shanghai, China). All cell lines had been authenticated in the past 3 years and again within 4 months after completion of the experiments. All cell lines had been tested for mycoplasma contamination frequently. Their identities were characterized by short tandem repeat analysis. All of these cell lines were cultured in high glucose Dulbecco's modified Eagle's medium supplemented by 10% fetal bovine serum, 100 units·mL^−1^ penicillin and 100 μg·mL^−1^ streptomycin (complete culture medium).

### Preparation of RCPs

The experimental protocol was reviewed and approved by the Animal Care and Research Committee of Xi'an Jiaotong University, China (SYXK:2023–004). All animal care procedures adhered to the guidelines for the ethical treatment and use of animals. Pregnant SD female rats with fetuses at post embryonic days 19–21 were killed by CO_2_ inhalation and subsequently cervical dislocation for the isolation of fetal rats [6]. The renal tissues were dissected from fetal rats. After removal of renal capsule, big blood vessels and connective tissues under a microscope on ice, the remaining renal tissues were cut into small pieces (approximately 1 mm^3^ in size), which were digested with four or five volumes of preheated 0.1%–0.2% (m/v) type IV collagenase and 0.05%–0.1% (m/v) type II collagenase (Life Technologies, Carlsbad, CA, USA) at 37 °C for 30 min, followed by stopped with complete culture medium RPM‐1640 and Dulbecco's modified Eagle's medium/F12 containing 10% fetal bovine serum and 1% dual antibiotics [7]. The digestive mixtures were sequentially passed through 80, 100 and 400 mesh filters, and the filtrates were centrifuged. The pelleted cells were resuspended in a complete culture medium and cultured in a flask that had been coated with 3–5 μg·cm^−2^ type I mouse tail collagen. After culture for 12 h at 37 °C in a 5% CO_2_ incubator, the cell samples were observed under a microscope.

RC samples (2 × 10^7^ cells per tube) were randomized and digested with 0.24% trypsin (pH 8.0; Beyotime, Shanghai, China), 4% alkaline protease (pH 8.5; Beyotime), 1.5% papain (pH 7.0; Beyotime) or 0.43% protamex (pH 7.0; Novozymes, Copenhagen, Denmark) in 50 mL of phosphate‐buffered saline at 45 °C for 4 h, followed by inactivating at 85 °C in a water bath for 10 min and centrifuged. Some digested samples were frozen and stored at −20 °C.

The remaining digested samples were suspended in 5 mL of solvent and centrifuged at 7155 ***g*** for 1 min to remove big pieces. The different samples were sequentially subjected to ultrafiltration through tubes with a molecular mass of 10 kDa and 3‐kDa filters using a CO_2_ pressure < 0.25 Mpa. The obtained fragmental samples with a molecular mass ≥ 10, 10–3 or < 3 kDa were processed into lyophilization or used for subsequent experiments. The concentrations of individual samples were measured by the BCA assay using a specific kit (Thermo Fisher Scientific, Waltham, MA, USA) and quantified, according to a standard curve established using different concentrations of bovine serum albumin.

### Cell counting kit‐8 (CCK)‐8 assay

MCF‐7 cells (1 × 10^4^ cells per well) were treated in triplicate with different doses of each type of RC extract in 96‐well plates for varying time periods. The negative control cells were treated with the diluent of physiological saline, whereas positive control cells were treated with different concentrations (0.1–5 mm) of 5‐fluorouracil (5‐FU) (Leica, Wetzlar, Germany). During the last hour of culture, each well of cells was treated with 10 μL of CCK‐8 solution. The absorbance of the supernatants of individual wells was measured at 450 nm using a microplate reader. Similarly, the effect of different synthesized RCPs (5–45 μg·mL^−1^) on the viability of different types of human cancer cells was tested with CCK‐8 assays using the diluent of physiological saline containing the same concentration of dimethylsulfoxide as a negative control and the same concentrations of 5‐FU as the positive controls. Ten RCPs with a predicted strong bioactivity were synthesized by GenScript (Illumina, San Diego, CA, USA) and their purity, sequences and solubility are shown in Table 1. After treatment for 48 h, the *in vitro* cytotoxicity of different concentrations of each peptide against each type of cancer cell was determined and the half maximal inhibitory concentration (IC_50_) of each type of RCP against each type of cancer cells was calculated using prism (GraphPad Software Inc., San Diego, CA, USA). The formula used to calculate the cytotoxicity rate of each concentration of RCP was: Cytotoxicity rate (%) = (absorbance value of the control group − absorbance value of the experimental group)/absorbance value of the control group × 100%. In addition, MCF‐7 cells were treated with each type of RCP at 45 μg·mL^−1^ for 12–72 h and the dynamic impact of RCP treatment on the viability of MCF‐7 cells was determined every 12 h using CCK‐8 assays.

**Table 1:** The synthesized and characterized peptides.

Name	Order ID	Sequence	Molecular mass	Length	Solubility	HPLC	Homology	Protein names	Method
RCP‐1	C8537MRWG0–1	QQHMKFGKKCWRKVWALLYA	2522.06	20	0.1% Dimethylsulfoxide	95.10%	32%	Docking protein 3 (Downstream of tyrosine kinase 3)	Trypsin digested
RCP‐2	C8537MRWG0–4	SSGCPPLPPRRGAPAGWLSH	2043.32	20	Water, pH 7.0	97.30%	6%	Brain‐derived neurotrophic factor, brain‐derived neurotrophic factor	Trypsin digested
RCP‐3	C8537MRWG0–7	GPAGPQGPR	835.91	9	Water, pH 7.0	96.40%	12%	Collagen alpha‐1 (I) chain	Trypsin digested
RCP‐4	C8537MRWG0–10	FAGDDAPR	847.88	8	Water, pH 7.0	98.60%	100%	Actin, cytoplasmic 2	Trypsin digested
RCP‐5	C8537MRWG0–13	VCMRVCRTWSRWCYDKRLWP	2645.18	20	0.1% Dimethylsulfoxide	95.70%	28%	F‐box and leucine‐rich repeat protein 19 (F‐box and leucine‐rich repeat protein 19 (Predicted), isoform CRA_a)	Trypsin digested
RCP‐6	C8537MRWG0–16	EWLKVPCLSTRLINPENMGF	2347.77	20	0.1% Dimethylsulfoxide	96.50%	39%	Sacsin molecular chaperone	Trypsin digested
RCP‐7	C8537MRWG0–19	RFFESFGDL	1117.22	9	0.1% Dimethylsulfoxide	98.40%	100%	GLOBIN domain‐containing protein	Protamex‐digested
RCP‐8	C8537MRWG0–22	GIYAPDSPRAF	1193.31	11	0.1% Dimethylsulfoxide	98.20%	97%	Vanin 1	Protamex‐digested
RCP‐9	C8537MRWG0–25	PAPPKPEPK	960.13	9	Water, pH 7.0	97.40%	100%	Non‐histone chromosomal protein HMG‐17	Trypsin digested
RCP‐10	C8537MRWG0–28	IYAPDSPRAF	1136.26	10	Water, pH 7.0	95.50%	97%	Vanin 1	Protamex‐digested

### LC‐MS/MS peptide identification

According to the results of the CCK‐8 experiment, peptide samples from trypsin, papain and protamex groups were purified by capillary HPLC. Briefly individual digested samples were loaded using an automatically sample loading device on the C18 sample column (100 μm × 20 mm with 5 μm particles; Thermo Fisher Scientific), which was connected to the Easy‐capillary separating C18 column (75 μm × 10 cm with 3 μm particles; Thermo Fisher Scientific). The columns were equilibrated with 95% A solution (0.1% formic acid) and samples were run at 250 nL·min^–1^ from 0 to 50 min with linearly increased concentrations of 0–35% B solution (0.1 formic acid, 84% acetonitrile); from 50 to 68 min with 35–100% B solution and maintaining with 100% B solution for another 2 min. Subsequentially, the purified peptides were identified by tandem MS analysis using a QExactive mass spectrometer (Thermo Fisher Scientific) for 60 min with positive iron detection. The mass to charge ratios (*m/z*) of peptides and peptide fragments were collected using 10 fragment maps (MS2 scan) after each full scan. The mass spectrometry test raw file (RawFile) was retrieved from the corresponding database using MaxQuant 1.5.5.1 software and their amino acid sequences were obtained based on the *m/z* values of individual peptide fragments, with their quantitative amounts being determined using the software supplied. The deducted amino acid sequences of all peptides were searched against database to confirm the sequences of amino acids in those peptides. The relevant parameters and descriptions used for database search are shown in Table S1. The biological activities of individual peptides were predicted using peptideranker (http://distilldeep.ucd.ie/peptideranker) with a threshold of 0.5 for training.

### Statistical analysis

Data are expressed as the mean ± SEM. The data distribution was analyzed by Shapiro–Wilk test. The IC_50_ values of each peptide for individual cell lines were determined using the prism, version 8 (GraphPad Software Inc.). Briefly, all dose–response data were converted into binary categorical variables, which were automatically analyzed by prism for non‐linear regression to determine and report the IC_50_ and its SD for each peptide in a specific cell line. The difference among groups was analyzed by analysis of variance and post‐hoc Tukey's tests and the comparison between groups was performed using Student's *t*‐test and prism, version 8 (GraphPad Software Inc.). *P* < 0.05 was considered statistically significant.

## Results

### Digestion of RCs with trypsin, protamex and papain, but not alkaline, protease results in bioactive components with cytotoxicity against MCF‐7 cells

**Fig. 1:**
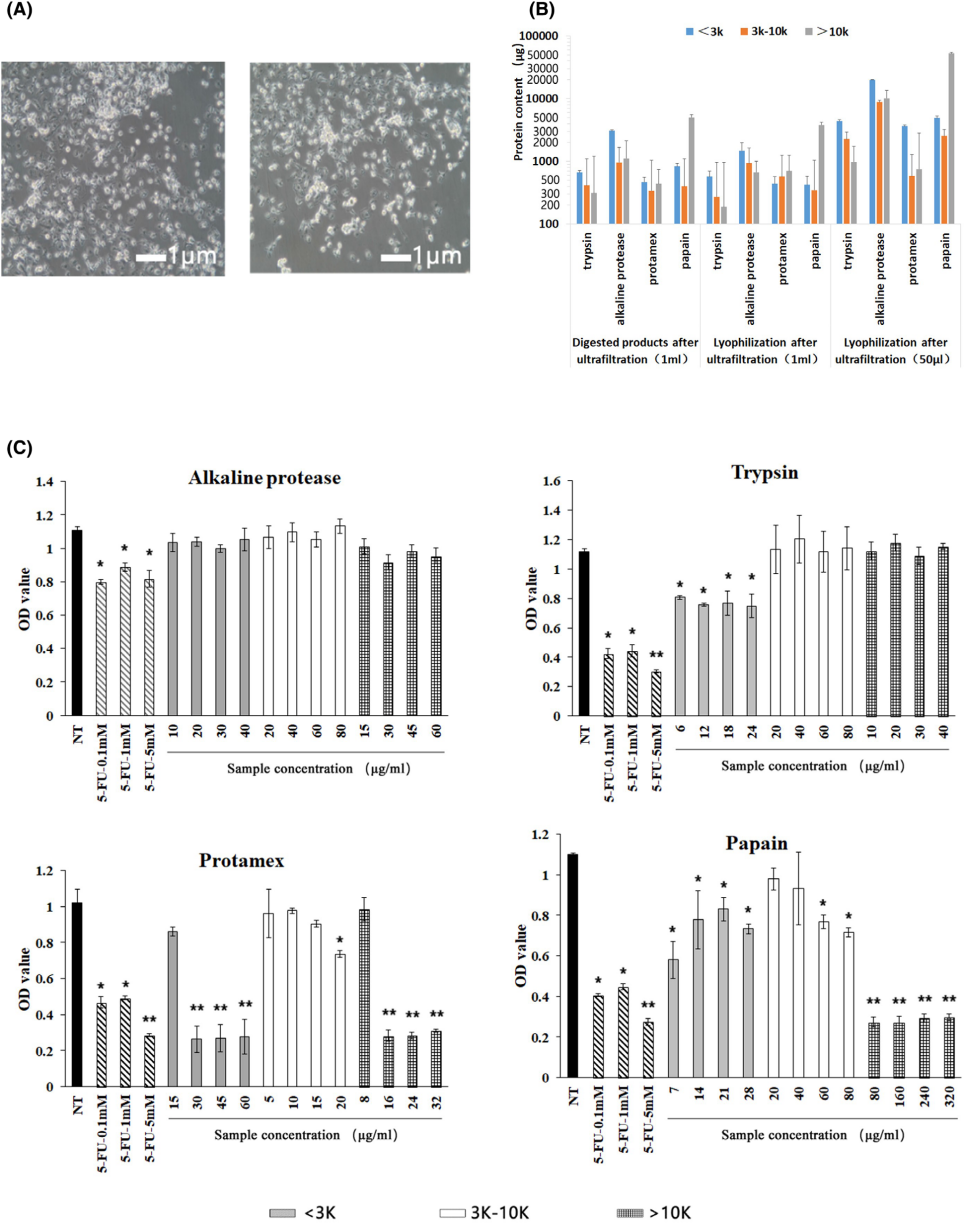
Preparation of RC and bioactive components, and analysis of their cytotoxicity against MCF‐7 cells. (A) Morphological observation of cultured primary RCs after culture for 72 h by microscopy (magnificaiton × 100; scale bar = 1 μm) (B) Protein contents of trypsin, alkaline protease, protamex and papain‐digested products after filtrations and lyophilization (50 μL and 1 mL). (C) Preliminarily testing the cytotoxicity of different components of RC products against MCF‐7 cells *in vitro* using negative control (NT) and positive control (5‐FU). *P < 0.05 and **P < 0.01 vs. the NT group, as determined by Student's *t*‐test. Error bars represent the mean ± SEM; *n* = 3 technical replicates.

To explore possible bioactive compounds in RCs, we dissected renal tissues from embryonic SD rats at post embryonic days 19–21 and digested the renal tissues with collagenase for 30 min. The digested tissue samples were filtered and centrifuged. The isolated RCs were cultured and observed under a microscope. The RCs displayed circular or spherical shapes, with intact cell membranes, regular morphology, strong adhesion ability and good growth status (Fig. 1A). After culture, they proliferate with time. The RC samples were randomized and digested with trypsin, alkaline protease, protamex or papain for 4 h. The protein concentrations of digested products were 1008.91 ± 32.38, 5379.61 ± 259.85, 577.86 ± 19.19 and 5603.6 ± 301.26 μg·mL^−1^, respectively. Further ultrafiltration through different sizes of filters and lyophilization, the total protein contents increased to 7623.3 ± 209.53, 38850.67 ± 1290.11, 4978.68 ± 6369.19 and 60158.3 ± 7723.85 μg per 50 μL, respectively. Quantitative analysis revealed the contents of different sizes of proteins and peptides in the digested products (Fig. 1B). The high contents of peptides with a molecular mass < 3 kDa existed in the alkaline protease‐digested RCs, whereas similar percentages of peptides with a molecular mass < 3 kDa were present in the other samples. It was notable that there were high percentages of proteins with a molecular mass > 10 kDa in the papain‐digested products. Functionally, we tested the cytotoxicity of different concentrations of each group of the digested products against MCF‐7 cells by CCK‐8 assays. As expected, treatment with positive control of 5‐FU (0.1–5 mm) significantly reduced the viability of MCF‐7 cells in a dose‐dependent manner (*P* < 0.01), whereas treatment with different doses of each group of alkaline protease‐digested products did not significantly affect the viability of MCF‐7 cells in our experimental condition (*P* > 0.05) (Fig. 1C). Furthermore, treatment with 30–60 μg·mL^−1^ < 3 kDa, 20 μg·mL^−1^ < 3–10 kDa or 16–32 μg·mL^−1^ > 10 kDa protamex‐digested products dramatically decreased the viability of MCF‐7 cells. Similarly, treatment with 80–320 μg·mL^−1^ > 10 kDa, 7–28 μg·mL^−1^ < 3 kDa papain‐digested products or 6–24 μg·mL^−1^ < 3 kDa trypsin‐digested products, but not other concentrations of products, significantly reduced the viability of MCF‐7 cells (*P* < 0.05 for all). Collectively, digestion of RCs with different enzymes led to different components of products, and the papain‐digested, trypsin‐digested and protamex‐digested, but not the alkaline‐digested, < 3 kDa RC products had potent cytotoxicity against MCF‐7 cells *in vitro*.

### Identification of bioactive peptides in the enzyme‐digested RC products

**Fig. 2:**
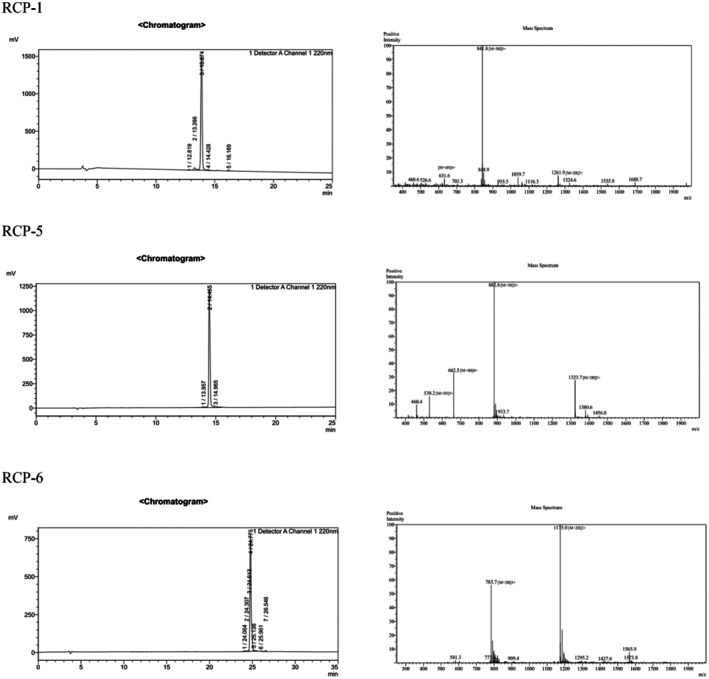
HPLC and MS analyses of RCP‐1, RCP‐5 and RCP‐6; *n* = 3 technical replicates.

Given that treatment with < 3 kDa papain‐digested, trypsin‐digested and protamex‐digested RC products significantly decreased the viability of MCF‐7 cells, we characterized bioactive components in these products by HPLC‐MS analyses. We identified 22 peptides in the papain‐digested products, 30 peptides in the trypsin‐digested products and 43 peptides in the protamex‐digested products (Table S1). Subsequently, the potential bioactivity of individual peptides were predicted using peptideranker (http://distilldeep.ucd.ie/peptideranker). There were 10 peptides predicted with strong bioactivity, based on a peptideranker value > 0.5, and they were selected, synthesized and characterized (Fig. 2 and Table 1).

### RCPs 1, 5 and 6, but not others, have a potent cytotoxicity preferably against different types of cancer cells

**Fig. 3:**
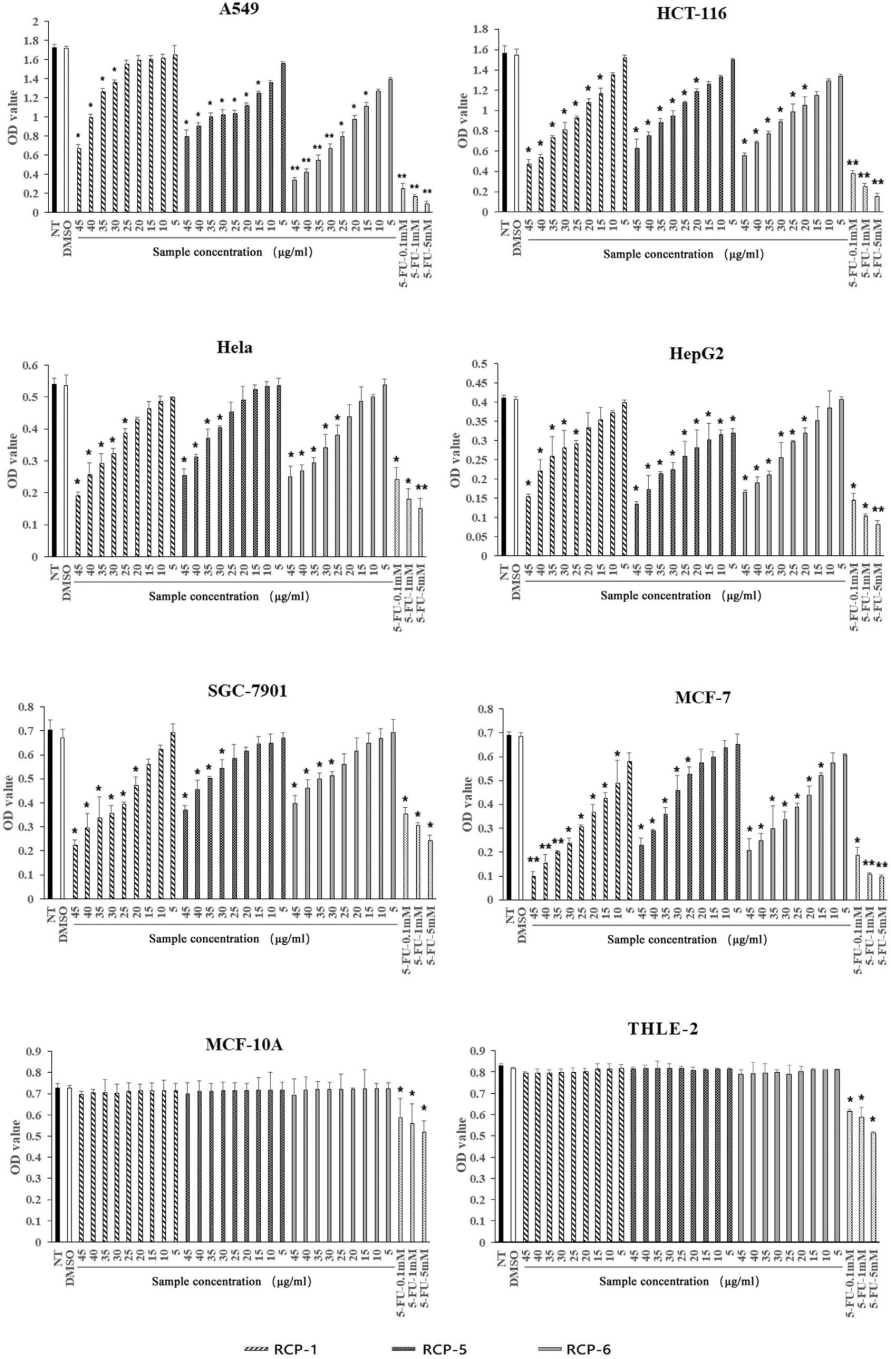
The cytotoxicity of RCP‐1, RCP‐5 and RCP‐6 preferably against human cancer cells. The indicated cell lines were treated in triplicate with negative control, positive control of 5‐FU, or different doses of RCP‐1, RCP‐5 or RCP‐6 for 48 h. The viability of cells in individual wells was measured by CCK‐8 assays. Data are expressed as the mean ± SEM of each group for three separate experiments. *P < 0.05 and **P < 0.01 vs. the NT group, as determined by Student's *t*‐test; *n* = 3 technical replicates.

**Fig. 4:**
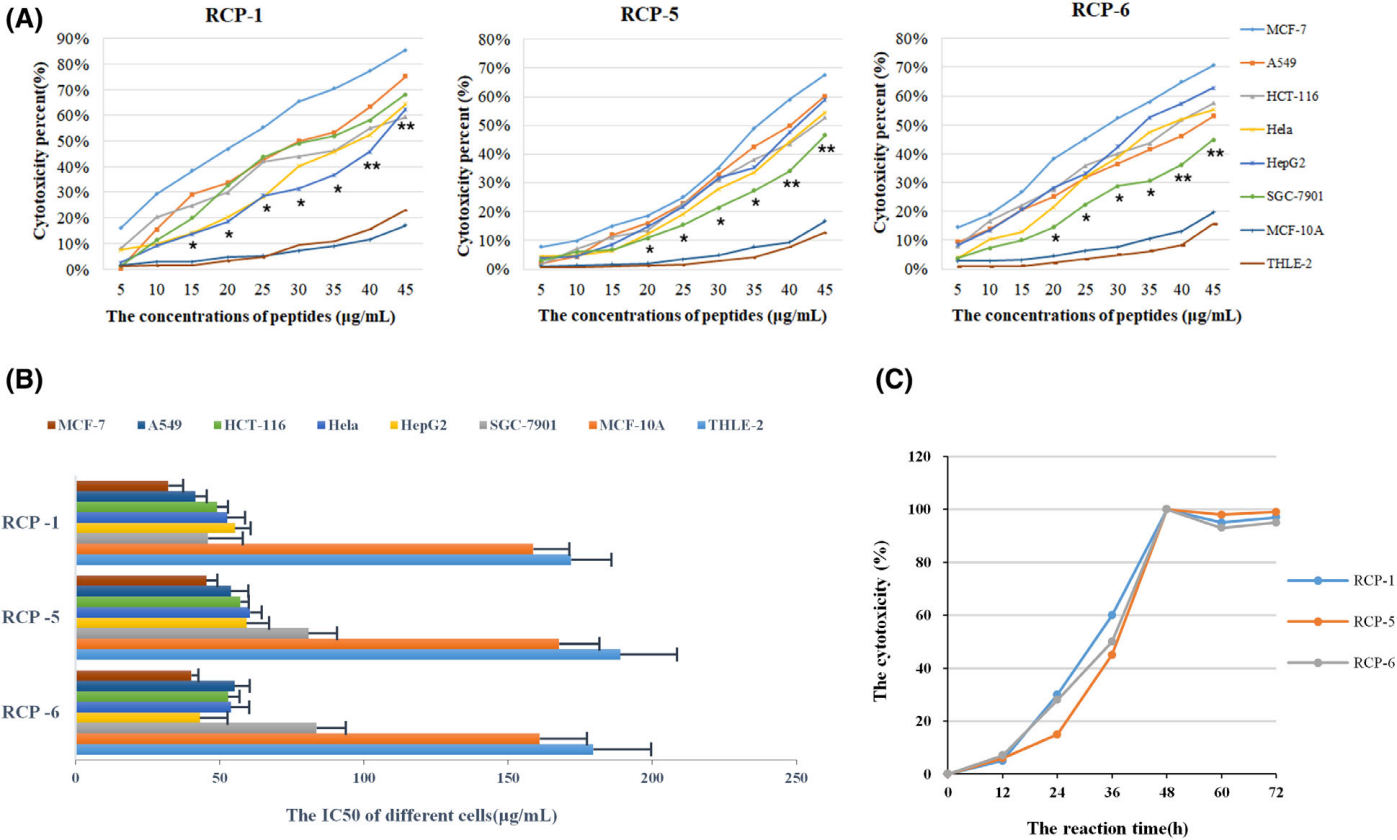
The time‐ and dose‐dependent cytotoxicity of RCP‐1, RCP‐5 and RCP‐6 preferably against human cancer cells. (A) The dose‐dependent cytotoxicity of each peptide against cancer cells. *P < 0.05 and **P < 0.01 vs. the MCF‐10A or THLE‐2 cells, as determined by Student's *t*‐test. (B) The IC_50_ values of each peptide against the indicated cell lines. (C) The dynamic cytotoxicity of individual peptides against MCF‐7 cells increased with time from 12 to 48 h post treatment and maintained at the highest levels throughout the observation period of 72 h. Error bars represent the mean ± SEM; *n* = 3 technical replicates.

To test the cytotoxicity against malignant tumors, human different types of cancer cells, including MCF‐7, A549, HCT‐116, Hela, HepG2, SGC‐7901 and non‐tumor control MCF‐10A and THLE‐2 cells were treated with each peptide at 5–45 μg·mL^−1^ for 48 h using the diluent containing the same concentration of dimethylsulfoxide as a negative control. The cell viability was tested by CCK‐8 assays. As shown in Fig. 3, treatment with the positive control of 5‐FU, but not the negative control, significantly decreased the viability of non‐tumor MCF‐10A and THLE‐2 cells (*P* < 0.05 for all). Similarly, treatment with different doses of RCP‐2, 3, 4, 7, 8, 9 and 10 did not preferably decrease the viability of tumor cells, and rather decreased the viability of both cancer (MCF‐7, A549, HCT‐116, Hela, HepG2 and SGC‐7901) and non‐tumor (MCF‐10A and THLE‐2) cells (Fig. S1). By contrast, treatment with different doses of RCP‐1, RCP‐5 or RCP‐6, similar to different doses of 5‐FU, significantly decreased the viability of these cancer cells, although only treatment with the highest doses of them slightly reduced the viability of non‐tumor MCF‐10A and THLE‐2 cells, highlighting that RCP‐1, RCP‐5 or RCP‐6 had potent cytotoxicity preferably against cancer cells. Indeed, the cytotoxicity of RCP‐1, RCP‐5 or RCP‐6 at a concentration ≥ 15–20 μm against non‐tumor MCF‐10A and THLE‐2 cells was statistically lower than tumor cell lines (*P* < 0.05 or *P* < 0.01). Quantitative analyses revealed that the cytotoxicity of these RCPs increased with elevated concentrations of the peptide, indicating that the cytotoxicity of different concentrations of the RCP against cancer cells was dose‐dependent (Fig. 4A). The IC_50_ values of individual RCPs are shown in Fig. 4B. Further testing of the dynamic effect of RCP‐1, RCP‐5, or RCP‐6 at the highest dose (45 μg·mL^−1^) revealed that the cytotoxicity of individual peptides against MCF‐7 cells increased with time from 12 h post treatment to 48 h and was maintained at the highest levels throughout the observation period of 72 h (Fig. 4C). Collectively, these data demonstrate that RCP‐1, RCP‐5 and RCP‐6 have a potent cytotoxicity preferably against cancer cells in a dose‐dependent manner.

## Discussion

Traditional chemotherapeutic drugs usually have severe side effects and unsatisfactory therapeutic efficacy [8, 9, 10, 11, 12]. Recent studies have shown that some bioactive peptides have potent antitumor activity against malignant tumors with a high safety profile [13, 14, 15, 16, 17]. These small peptides usually have < 50 amino acids [18, 19, 20, 21, 22]. Currently, some peptide drugs have been applied in anti‐tumor clinical treatment [23, 24, 25, 26, 27] and they can be derived from different resources, such as plants and animals, or synthesized [28, 29, 30, 31, 32, 33, 34, 35, 36, 37]. In the present study, we identified several bioactive RCPs with a potent cytotoxicity preferably against different types of human malignant tumor cells *in vitro* in a dose‐dependent manner. Hence, our findings may provide a new basis for the design of new bioactive peptides for the treatment of solid cancer in the clinic.

The methods for preparing bioactive peptides from animal tissues mainly include organic solvent extraction, enzyme lysis, physical homogenization and ultrasonic extraction. Organic solvent extraction can use two incompatible reagents with different distribution coefficients to obtain the active components of animal proteins and peptides. Although this method is easily performed at low cost, and it is easy to recover the bioactive peptides in that the organic solvents usually denature and inactivate proteins, it does require a low temperature environment, such that it is difficult to control with a low extraction rate [38]. The lysis method can rapidly rupture cells at low cost, with a high extraction rate, but the solvent is toxic, easy to denature and inactivates proteins, leading to subsequent complicated processes for purification [39]. Physical homogenization can use mechanical and hydraulic shear to break down tissue cells and release bioactive peptides, but the bioactive peptide production rate is low [40]. Ultrasonic extraction uses the ultrasonic radiation pressure generated by the strong cavitation reaction, as well as mechanical vibration, disturbance, high acceleration, emulsification, diffusion, crushing and stirring, and other multi‐stage effects, to increase the frequency of molecular movement and speed of the substance, increasing the penetration of the solvent, leading to target components in the solvent. Although the ultrasonic extraction can result in a high product rate, low cost and simple operation it can inactivate and denature proteins and the ultrasound‐related cavitation can produce reactive oxygen species, complicating the biological characterization of bioactive peptides [41]. Enzymatic hydrolysis can use active enzymes to hydrolyze specific substances with high yields of bioactive peptides [42] and has been widely used for preparing bioactive peptides from animal tissues [43]. In the present study, we first employed collagenases to digest fetal kidneys to prepare RCs and, subsequently, we used four different types of enzymes to prepare RCPs. We found that digestion with either trypsin, papain, protamex or alkaline protease led to some percentages of peptides with a molecular mass < 3 kDa. Interestingly, the trypsin, papain or protamex‐digested < 3 kDa products, but not the alkaline protease‐digested < 3 kDa products, had a potent cytotoxicity against human breast cancer MCF‐7 cells. Furthermore, treatment with 30–60 μg·mL^−1^ < 3 kDa, 20 μg·mL^−1^ < 3–10 kDa or 16–32 μg·mL^−1^ > 10 kDa protamex‐digested products dramatically decreased the viability of MCF‐7 cells. Similarly, treatment with 80–320 μg·mL^−1^ > 10 kDa, 7–28 μg·mL^−1^ < 3 kDa papain‐digested products or 6–24 μg·mL^−1^ of < 3 kDa trypsin‐digested products, but not other concentrations of products, significantly reduced the viability of MCF‐7 cells. Collectively, digestion of RCs with different enzymes led to different components of products with potent cytotoxicity against MCF‐7 cells *in vitro*. Thus, the digestion with trypsin, papain or protamex is a useful method for the preparation of bioactive RCPs from fetal rats. The lack of bioactivity of the alkaline protease‐digested < 3 kDa products may stem from the broad substrate specificity of alkaline protease, leading to small peptides without biological activity.

To explore the nature of bioactive RCPs, we identified RCPs using HPLC‐MS and found 22 RCPs in the papain‐digested products, 30 RCPs in the trypsin‐digested products and 43 RCPs in the protamex‐digested products. These RCPs were derived from different conservative proteins in varying species, including in humans. These novel findings are the first to identify the nature of RCPs and may help in design of bioactive peptide‐based medicines. Subsequently, we predicted the potential biological activity using peptideranker (http://distilldeep.ucd.ie/peptideranker) and found 10 RCPs with bioactivity greater than a threshold of 0.5. Interestingly, these RCPs were derived from docking protein 3, brain‐derived neurotrophic factor, collagen alpha‐1(I) chain, actin cytoplasmic 2, F‐box and leucine‐rich repeat protein 19, sacsin, GLOBIN domain‐containing protein, vanin 1 and HMG‐17. More importantly, we discovered three RCPs (RCP‐1, RCP‐5 and RCP‐6) with potent cytotoxicity preferably against different types of malignant tumor cells in a dose‐dependent manner. Notably, RCP‐1, RCP‐5 and RCP‐6 were 20 amino acids in length and generated from trypsin‐digested RCs. However, the remaining seven peptides were relatively shorter (RCP‐3, RCP‐4, RCP‐7, RCP‐8, RCP‐9 and RCP‐10) or contained several prolines, leading to unique rigidity and peptide folding. These characteristics may affect their biological antitumor activity, although the bioactivity of these peptides was also predicted. RCP‐1, RCP‐5 and RCP‐6 had a low aqueous solubility and their sequences had no obvious similarity, suggesting that their antitumor activities may occur through different mechanisms. Moreover, these RCPs were derived from docking protein 3, F‐box and leucine‐rich repeat protein 19 and sacsin, respectively. RCP‐1, RCP‐5 and RCP‐6 had a moderate homology with the sequences of corresponding human proteins (Table 1). Given that these proteins are expressed conservatively in different organs of different species of animals, we aim to further investigate whether these peptides are naturally present in the circulating system of humans. Evidently, the IC_50_ values of these RCPs for cancer cells were two‐ to three‐fold lower than that for non‐tumor cells in our experimental system. The preference of RCPs against cancer cells suggests that treatment with those peptides in an optimal dose may have few adverse effects. The cytotoxicity against different types of cancer cells indicates that these RCPs may have antitumor activities against broad types of malignant tumors. We are interested in further investigating the pharmacological action of these RCPs in the cytotoxicity against different types of cancers.

In summary, the results of the present study indicate that RCs were effectively digested with trypsin, papain or protamex. These digested products were sequentially filtrated into different sizes of components to enrich and prepare bioactive RCPs. Furthermore, these RCPs were derived from conservative proteins in the renal tissues, as well as many other organs, in different species. More importantly, RCP‐1, RCP‐5 and RCP‐6 had a potent cytotoxicity preferably against different types of cancer cells in a dose‐dependent manner. Therefore, our novel findings may provide a basis for the design of bioactive peptide‐based therapies for different types of solid cancer.

## Conflicts of interest

The authors declare that they have no conflicts of interest..

## Author contributions

YC performed calculation processing. LS conceived and supervised the study. JW performed HPLC and MS analysis. JD performed the CCK8 experiments. YZ and YY provided the SD rat materials and cells. ZZ drafted the manuscript. All authors have read and approved the final version of the manuscript submitted for publication.

## Supporting information


**Figure S1.** (A) The dose‐dependent cytotoxicity of RCP‐2, RCP‐3, RCP‐4, RCP‐7, RCP‐8, RCP‐9 and RCP‐10 preferably against human cancer cells. (B) HPLC and MS of RCP‐2, RCP‐3, RCP‐4, RCP‐7, RCP‐8, RCP‐9 and RCP‐10.

**Table S1.** The peptides of protamex, papain and trypsin groups.

## Data Availability

The data included in this study are available from the corresponding author upon reasonable request.
