# Supplementary figures and images for: Preparation and characterization of renal cell peptides from fetal rats for their antitumor activity
Source: FEBS Open Bio. 2025 Jun 26;15(11):1841–53. doi: 10.1002/2211-5463.70075 (PMC12582985; doi:10.1002/2211-5463.70075)

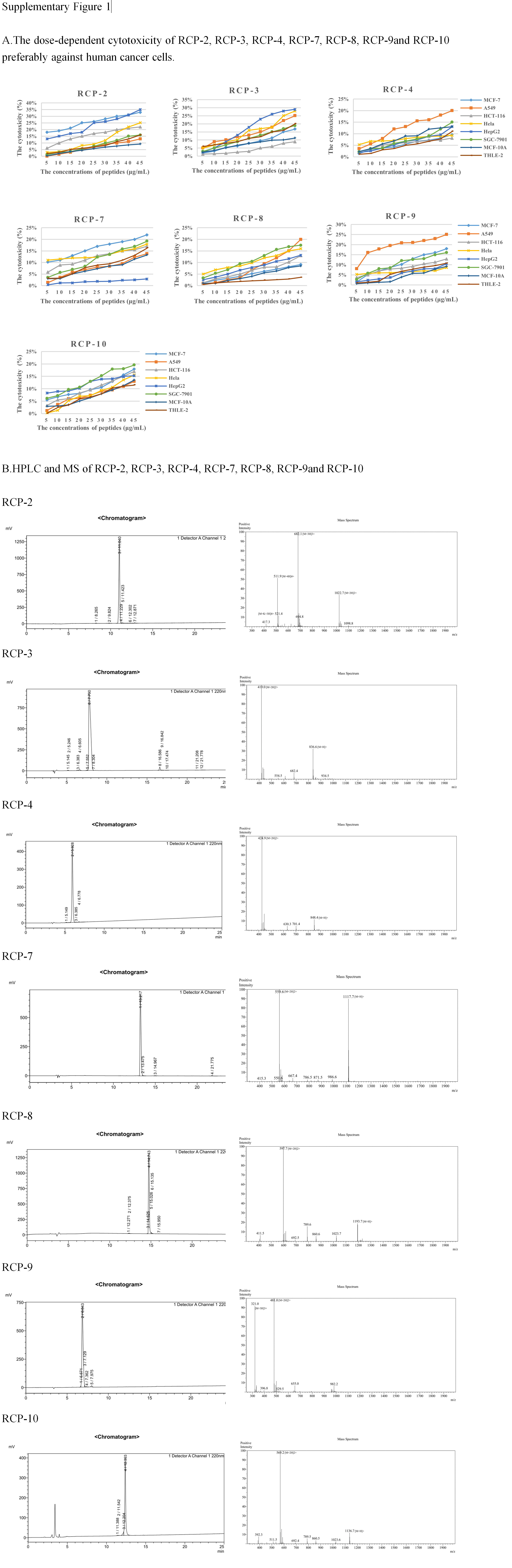

Supplement: Supplementary file 1 — Figure S1. (A) The dose‐dependent cytotoxicity of RCP‐2, RCP‐3, RCP‐4, RCP‐7, RCP‐8, RCP‐9 and RCP‐10 preferably against human cancer cells. (B) HPLC and MS of RCP‐2, RCP‐3, RCP‐4, RCP‐7, RCP‐8, RCP‐9 and RCP‐10. [file FEB4-15-1841-s001.tif]
