# Supplementary material for: Preparation and characterization of renal cell peptides from fetal rats for their antitumor activity
Source: FEBS Open Bio. 2025 Jun 26;15(11):1841–53. doi: 10.1002/2211-5463.70075 (PMC12582985; doi:10.1002/2211-5463.70075)
Supplement: Supplementary file 2 — Table S1. The peptides of protamex, papain and trypsin groups. [file FEB4-15-1841-s002.docx]

Supplementary Table 1. The peptides of protamex, papain, and trypsin groups

| Sequence | Length | Mass | Leading razor protein | Protein names | Gene Names | Start position | End position | Unique (Groups) | Charges | Score | group |
| --- | --- | --- | --- | --- | --- | --- | --- | --- | --- | --- | --- |
| ADSPPQDHS | 9 | 952.38864 | D3ZFH5 | Inter-alpha-trypsin inhibitor heavy chain 2 | Itih2 | 645 | 653 | yes | 2 | 101.28 | protamex group |
| APLVTPASKDLACLSRAPTGMFRQWSVQSGPAPRRSESQA | 40 | 4253.1586 | A0A8I6ACB7 | A0A8I6ACB7 |  | 15 | 54 | yes | 4 | 14.296 | protamex group |
| CDGFQDCEDGQDEQNCTRSIPCTNRTFKCGNDICFRKQNAQCDGI | 45 | 5075.0878 | R9PXY0; P86091 | R9PXY0 | Tmprss7 | 524 | 568 | yes | 5 | 0.90742 | protamex group |
| DAEDGHSPGEQQ | 12 | 1268.4905 | Q6MG74 | Complement factor B, EC 3.4.21.47 (C3/C5 convertase) | Cfb Apom Bat4 Bf C2 C4a Csnk2b G7e Hspa1a Hspa1b Hspa1l Ly6g5c Ly6g6c Neu1 Ng35 Rps25-ps2 Stk19 | 243 | 254 | yes | 2 | 135.83 | protamex group |
| DVFLGTFLYEYSR | 13 | 1608.7824 | A0A0G2JSH5 | Albumin | Alb | 348 | 360 | yes | 2 | 138.71 | protamex group |
| EELNADLFRGTLDPVE | 16 | 1816.8843 | A0A8I6AQL9 | Similar to heat shock protein 8 | LOC688932 | 225 | 240 | yes | 2 | 62.466 | protamex group |
| ELNRVIQR | 8 | 1026.5934 | A0A0G2K509 | Keratin, type II cytoskeletal 1 (Cytokeratin-1) (Keratin-1) (Type-II keratin Kb1) | Krt5 Krt1 LOC102551453 | 399 | 406 | yes | 2 | 117.18 | protamex group |
| ESSKKKPQAEEESKEALKTSEHCEKEKASSKDLKHTHGK | 39 | 4406.2136 | A0A8I6A9H5; A0A8I6A1A3; M0RC54 | A0A8I6A9H5 | Bod1l1 | 583 | 621 | yes | 5 | 12.234 | protamex group |
| FAGDDAPR | 8 | 847.38243 | A0A8I6AQR0 | Actin, cytoplasmic 2 | Actg1 | 21 | 28 | yes | 2 | 116.54 | protamex group |
| GFAGDDAPRA | 10 | 975.44101 | A0A8I6AQR0 | Actin, cytoplasmic 2 | Actg1 | 20 | 29 | yes | 2 | 119.29 | protamex group |
| GIYAPDSPRA | 10 | 1045.5193 | Q4KLZ0 | Vanin 1 | Vnn1 rCG_41942 | 280 | 289 | yes | 2 | 107.9 | protamex group |
| GIYAPDSPRAF | 11 | 1192.5877 | Q4KLZ0 | Vanin 1 | Vnn1 rCG_41942 | 280 | 290 | yes | 2 | 124.98 | protamex group |
| GKETSPNRQLRRCPGSHCLTITDVPITVYATMRKPPAQ | 38 | 4221.1722 | B0BND4 | B0BND4 | Fam229b | 35 | 72 | yes | 4 | 17.229 | protamex group |
| GPAGPQGPR | 9 | 835.43005 | A0A8I5ZRN2 | Collagen alpha-1(I) chain | Col1a1 | 990 | 998 | yes | 2 | 118.01 | protamex group |
| HEKTPVSEK | 9 | 1053.5455 | A0A0G2JSH5 | Albumin | Alb | 488 | 496 | yes | 2 | 156.35 | protamex group |
| HLDDLPGAL | 9 | 949.4869 | Q63910 | Alpha globin (Globin c1) (Hemoglobin alpha, adult chain 3) (RCG33691) | Hba-a3 Glnc1 GloA Hba-a1 Hba-a2 Hba2 LOC287167 rCG_33691 | 73 | 81 | yes | 2 | 118.06 | protamex group |
| HVKRWFLLLALLNSVMNPIIYSYKDEDMYNTMRKMICCAPHD | 42 | 5071.4866 | A0A8I6A9K7; Q8K5E0 | A0A8I6A9K7 | Lpar3 | 264 | 305 | yes | 6 | 3.3444 | protamex group |
| IIAPPERKYS | 10 | 1172.6554 | A0A8I6AQR0 | Actin, cytoplasmic 2 | Actg1 | 107 | 116 | yes | 2 | 99.139 | protamex group |
| IPAGIEVKVDDCNICHCHNGDWWKPAQCSKRECQGKQTV | 39 | 4395.0228 | D4A0X1; A0A8I6AU98 | D4A0X1 | Vwc2l | 137 | 175 | yes | 4 | 11.304 | protamex group |
| IYAPDSPR | 8 | 917.46068 | Q4KLZ0 | Vanin 1 | Vnn1 rCG_41942 | 281 | 288 | yes | 2 | 130.77 | protamex group |
| IYAPDSPRAF | 10 | 1135.5662 | Q4KLZ0 | Vanin 1 | Vnn1 rCG_41942 | 281 | 290 | yes | 2 | 119.41 | protamex group |
| KLRVDPVN | 8 | 939.55016 | Q63910 | Alpha globin (Globin c1) (Hemoglobin alpha, adult chain 3) (RCG33691) | Hba-a3 Glnc1 GloA Hba-a1 Hba-a2 Hba2 LOC287167 rCG_33691 | 91 | 98 | yes | 2 | 113.5 | protamex group |
| KLRVDPVNF | 9 | 1086.6186 | Q63910 | Alpha globin (Globin c1) (Hemoglobin alpha, adult chain 3) (RCG33691) | Hba-a3 Glnc1 GloA Hba-a1 Hba-a2 Hba2 LOC287167 rCG_33691 | 91 | 99 | yes | 2 | 111.01 | protamex group |
| LADSPPQDH | 9 | 978.44067 | D3ZFH5 | Inter-alpha-trypsin inhibitor heavy chain 2 | Itih2 | 644 | 652 | yes | 2 | 107.59 | protamex group |
| LADSPPQDHS | 10 | 1065.4727 | D3ZFH5 | Inter-alpha-trypsin inhibitor heavy chain 2 | Itih2 | 644 | 653 | yes | 2 | 106.91 | protamex group |
| LDDYLNGR | 8 | 964.46141 | A0A8I6A5C5 | Gelsolin (Actin-depolymerizing factor) (Brevin) | Gsn | 84 | 91 | yes | 2 | 120.65 | protamex group |
| LDEELGGTPVQSR | 13 | 1399.6943 | A0A8I6A5C5 | Gelsolin (Actin-depolymerizing factor) (Brevin) | Gsn | 462 | 474 | yes | 2 | 136.48 | protamex group |
| LNTGYQRQ | 8 | 978.48829 | Q5FX35 | Alpha 2 macroglobulin cardiac isoform |  | 41 | 48 | yes | 2 | 157.86 | protamex group |
| LPPNELEEYNQILLDMETTYSVANVCYTNCTCLSLEPDLTNIMAT | 45 | 5081.3252 | Q64603 | Q64603 |  | 13 | 57 | yes | 6 | 3.0239 | protamex group |
| LVVYPWTQRF | 10 | 1307.7026 | A0A8I5ZV52 | GLOBIN domain-containing protein |  | 33 | 42 | yes | 2 | 98.629 | protamex group |
| PDIKHHFPCKEVKESGHMFPSHLLVTATHMYCLREILSRK | 40 | 4714.3909 | A0A8I5YBN9; A0A8I6AAK0; D4AAH9 | A0A8I5YBN9 | Tbc1d23 | 570 | 609 | yes | 5 | 3.6024 | protamex group |
| QPLSVQWHWRPWTPCKTFAQRSLRRRQPRDGMPQCRDWK | 39 | 4917.4768 | A0A8I6GL85; A0A8I6AX70; G3V6B7; Q91ZT1 | A0A8I6GL85 | Flt4 | 415 | 453 | yes | 4 | 3.7052 | protamex group |
| RFFESFGDL | 9 | 1116.524 | A0A8I5ZV52 | GLOBIN domain-containing protein |  | 41 | 49 | yes | 2 | 110.31 | protamex group |
| RIYEDSDSAL | 10 | 1167.5408 | D3ZFC6 | Inter-alpha-trypsin inhibitor heavy chain 4 | Itih4 | 439 | 448 | yes | 2 | 163.9 | protamex group |
| RIYEDSDSALQ | 11 | 1295.5994 | D3ZFC6 | Inter-alpha-trypsin inhibitor heavy chain 4 | Itih4 | 439 | 449 | yes | 2 | 111.46 | protamex group |
| RVDPVNFK | 8 | 973.53451 | Q63910 | Alpha globin (Globin c1) (Hemoglobin alpha, adult chain 3) (RCG33691) | Hba-a3 Glnc1 GloA Hba-a1 Hba-a2 Hba2 LOC287167 rCG_33691 | 93 | 100 | yes | 2 | 113.91 | protamex group |
| TVLDSGISEVR | 11 | 1174.6194 | A0JN13 | Alpha-crystallin A chain | Cryaa rCG_60707 | 55 | 65 | yes | 2 | 144.09 | Papain  group |
| LADSPPQDH | 9 | 978.44067 | D3ZFH5 | Inter-alpha-trypsin inhibitor heavy chain 2 | Itih2 | 644 | 652 | yes | 2 | 107.59 | Papain  group |
| TLHQITELCGA | 11 | 1184.586 | A0A8I6G7F0; A0A0G2K285; A0A8I5ZKT6; A0A8I6AED1 |  |  | 75 | 85 | yes | 2 | 22.237 | Papain  group |
| RNECSAVYSKG | 11 | 1212.5557 | A0A8I6B4H4; A0A8I5ZUE5; A0A8I6ABJ6; A0A8I6A816; A0A8I6AN06; A0A8I5ZUJ3; D3ZC56; A0A8I5ZQD4 |  | Dst | 151 | 161 | yes | 2 | 23.071 | Papain  group |
| QGSAAPGNKDH | 11 | 1080.4948 | A0A8I5ZMA9; Q5FVN2 | Transmembrane protein 41B | Tmem41b | 28 | 38 | yes | 3 | 23.749 | Papain  group |
| WRPESFFDKVK | 11 | 1437.7405 | Q569A7; A0A8I5ZL86 |  | Dph5 | 140 | 150 | yes | 3 | 24.853 | Papain  group |
| LLQIANKIQ | 9 | 1039.639 | M9MMM9; A0A0G2JU82; A0A8I5ZUE8; A0A0G2JWA8; A0A8I6AP57; A0A0G2K9T4; A0A8I5ZYF6; A0A8I5YCC2; A0A8I6ATL8; A0A8I6AAP8; D3ZHV2 | Microtubule-actin cross-linking factor 1 | Macf1 | 387 | 395 | yes | 2 | 28.423 | Papain  group |
| DFSYLLSKV | 9 | 1070.5648 | D3ZCG0; A0A0G2K9F0; A0A8I6GLG6; A0A8I6A312; A7BJV7; A0A8I6AGI2; Q810T5 | Histone acetyltransferase; Histone acetyltransferase KAT7 | Kat7; HBO1 | 403 | 411 | yes | 2 | 29.149 | Papain  group |
| DLPALRKRAEI | 11 | 1280.7565 | A0A8I5Y087; Q5U2R8 |  | Mnda | 71 | 81 | yes | 2 | 30.57 | Papain  group |
| SICERVQTSAK | 11 | 1220.6183 | A0A8I5ZME2; A0A0G2K3H7; A0A8I6GHQ1 |  |  | 1020 | 1030 | yes | 2 | 32.841 | Papain  group |
| HLNLSQVQ | 8 | 937.49813 | F7EY07; Q498U5; A0A8I6GE65; A0A0G2K4W6; O88850 | Homeodomain-interacting protein kinase 3 | Hipk3 | 1028 | 1035 | yes | 2 | 40.946 | Papain  group |
| FVGDGFTR | 8 | 897.43447 | A0A8I5ZVN7; Q4KMB2; Q2I0Y6; Q9QYU7 | Ribosome biogenesis protein NSA2 homolog | Nsa2 | 114 | 121 | yes | 2 | 41.502 | Papain  group |
| AVVTSDGK | 8 | 775.40758 | A0A8I5ZV70; A0A0G2JTT6 |  |  | 4101 | 4108 | yes | 2 | 43 | Papain  group |
| PTSSLTQP | 8 | 829.41815 | A0A8I6AC36; M0RDF1; A0A8I5ZXX2 |  | Kdm3b | 529 | 536 | yes | 2 | 43.066 | Papain  group |
| SDQRENFP | 8 | 991.43592 | D3ZFP6 |  | Ticrr | 1051 | 1058 | yes | 2 | 44.721 | Papain  group |
| DFITNIDG | 8 | 893.41306 | F1M6I2; F1M6I1; A0A8I5ZY49 |  | LOC691033; LOC691044 | 553 | 560 | yes | 1 | 49.358 | Papain  group |
| VDFISDLIACLLQGC | 15 | 1608.7892 | A0A8I5ZRX4; F1LYI0 |  | Cecr2 | 53 | 67 | yes | 2 | 50.292 | Papain  group |
| QEPGAKEPMAE | 11 | 1185.5336 | A0A8I5ZZE5; G3V8N6; Q62798 | Dorsal root ganglia homeobox protein | Drgx | 98 | 108 | yes | 2 | 58.596 | Papain  group |
| RGAAGEQL | 8 | 800.41407 | A0A8I5ZQJ2; A0A8I5ZPT7; A0A8I6A3U0; A0A8I5ZUH0; A0A8I6ASG8; D4A8U7; A0A0G2K428; P28023 | Dynactin subunit 1 | Dctn1 | 509 | 516 | yes | 2 | 67.035 | Papain  group |
| ITGESGAGK | 9 | 818.4134 | F1LV10; A0A8I6AED4; A0A8I6G7W8; A0A8I6GLM5; A0A8I6AFE5; A0A8I6GGN1; A0A8I6ART5; A0A0G2K0F5; G3V8B0; F1M8F6; A0A0G2K1V4; G3V885; A0A8I6AFM0; A0A8I5Y7Z0; F1LMU0; A0A0G2K484; G3V6D8; B6RK61; G3V6E1; F1LRV9; A0A8I6AIQ9; Q62774; Q05096; P02564; P02563; Q29RW1; P12847 | Unconventional myosin-Ia; Unconventional myosin-Ib; Myosin-7; Myosin-6; Myosin-4; Myosin-3 | Myo1a; Myh7; Myh8; Myh6; Myh4; Myh3; Myh7b; Myh2; Myh1; Myo1b | 99 | 107 | yes | 2 | 81.296 | Papain  group |
| TVLDSGISEVR | 11 | 1174.6194 | A0JN13 |  | Cryaa | 55 | 65 | yes | 2 | 122.13 | Papain  group |
| LLLLLLLLL | 9 | 1035.7671 | Q91ZR6; A0A8I5ZU39; F1M0N4; H9C9P2; D3ZQQ8; A0A8I5ZPU0; A0A8I6GLV3; A0A8I6ADU9; A0A8I5ZPK7; D4ABB2; Q7TP75; A0A0G2JUV2; A0A8I5Y7D9; A0A0G2JWS6; A0A8I6GLR1; A0A8I5Y9Z3; A0A0G2K867; A0A8I6G4E1; Q6DQ95; G3V6E2; A0A8I5ZP92; Q9R104; A0A8I6GM86; A0A8I6ADB7; Q5I0I6; A0A8I5ZU03; A0A8I6G839; F7EZJ3; A0A8I6A0J7; A0A8I6AS10; Q9R105; A0A8I6AI14; Q6MG85; F8WSD3; F7EQT9; A0A8I6A1Z2; A0A8I5ZYQ2; A0A8I6GJM6; A0A8I6A469; Q67EV3; A0A8I5ZZ87; D4ADP7; A0A0G2JVH3; A0A8I6A8Y0; D4AD24; Q4G028; A0A0G2KB67; F1LRG0; M0RB68; D3ZGM7; A0A1W2Q642; A0A0G2JTR3; D3ZQJ8; A0A8I6G9L9; A0A8I6AE51; A0A8I6A6L0; A0A8I6A8W8; A0A8I5ZVL7; A0A0G2JVB2; A0A8I6GH61; A0A8I5ZM90; A0A8I6GFL9; A0A8I5ZTA7; D4AC02; A0A8I6ALZ7; D3ZE75; M0RB60; D4A3W2; D3Z981; A0A8I6AGW9; G3V928; A0A8I6A0U0; A0A5P8DHK3; A0A8I6AWY4; A0A8I5ZNW6; P0C172; P11384; Q9QZT3; Q9JHJ1; Q53AQ4; Q5RJL6; Q6P7C4; O35799; Q499S5; Q64562; Q5U367; Q91ZV2 | Phospholipase A(2); Beta-2-microglobulin; 1-acyl-sn-glycerol-3-phosphate acyltransferase; Tuberoinfundibular peptide of 39 residues; Secretin; Group 10 secretory phospholipase A2; Receptor activity-modifying protein 2; Transmembrane and ubiquitin-like domain-containing protein 1; Meteorin-like protein; Leucine-rich repeat-containing protein 26; Hereditary hemochromatosis protein homolog; Stromelysin-3; Steroid 21-hydroxylase; Procollagen-lysine,2-oxoglutarate 5-dioxygenase 3; Discoidin, CUB and LCCL domain-containing protein 2 | Slc35a4; Lrcol1; Mlc1; Ahsg; Pla2g10; Hfe; Ptprcap; Agpat1; Metrnl; Lilrb4; Tnfrsf25; Acpt; Syne2; Cyp21a1; LOC100912537; Pi16; Prss56; Scube2; Lrp8; Lrig1; Crb2; Plxna1; Lrp1; Pth2; Sct; Ramp2; Tmub1; Lrrc26; Mmp11; Cyp21; Plod3; Dcbld2 | 59 | 67 | yes | 2 | 132.11 | Papain  group |
| TVLDSGISEVR | 11 | 1174.6194 | A0JN13 | Alpha-crystallin A chain | Cryaa rCG_60707 | 55 | 65 | yes | 2 | 144.09 | Papain  group |
| LADSPPQDH | 9 | 978.44067 | D3ZFH5 | Inter-alpha-trypsin inhibitor heavy chain 2 | Itih2 | 644 | 652 | yes | 2 | 107.59 | Papain  group |
| TLHQITELCGA | 11 | 1184.586 | A0A8I6G7F0; A0A0G2K285; A0A8I5ZKT6; A0A8I6AED1 |  |  | 75 | 85 | yes | 2 | 22.237 | Papain  group |
| RNECSAVYSKG | 11 | 1212.5557 | A0A8I6B4H4; A0A8I5ZUE5; A0A8I6ABJ6; A0A8I6A816; A0A8I6AN06; A0A8I5ZUJ3; D3ZC56; A0A8I5ZQD4 |  | Dst | 151 | 161 | yes | 2 | 23.071 | Papain  group |
| QGSAAPGNKDH | 11 | 1080.4948 | A0A8I5ZMA9; Q5FVN2 | Transmembrane protein 41B | Tmem41b | 28 | 38 | yes | 3 | 23.749 | Papain  group |
| HAVSEGTK | 8 | 827.41373 | M0RBQ5 | Histone H2B | H2bu1 rCG_32940 | 110 | 117 | yes | 2 | 151.56 | Trypsin  group |
| TVLDSGISEVR | 11 | 1174.6194 | A0JN13 | Alpha-crystallin A chain | Cryaa rCG_60707 | 55 | 65 | yes | 2 | 144.09 | Trypsin  group |
| LLEGEESR | 8 | 931.46108 | G3V8C3 | Vimentin | Vim | 403 | 410 | yes | 2 | 143.85 | Trypsin  group |
| DVFLGTFLYEYSR | 13 | 1608.7824 | A0A0G2JSH5 | Albumin | Alb | 348 | 360 | yes | 2 | 138.71 | Trypsin  group |
| HTDAAPIIPN | 10 | 1047.5349 | Q9QUN4 | Pancreatic lipase |  | 220 | 229 | yes | 2 | 127.84 | Trypsin  group |
| GPAGPQGPR | 9 | 835.43005 | A0A8I5ZRN2 | Collagen alpha-1(I) chain | Col1a1 | 990 | 998 | yes | 2 | 118.01 | Trypsin  group |
| EGIPPDQQR | 9 | 1038.5094 | A0A8I6AN99 | RCG50143 (Ubiquitin-40S ribosomal protein S27a-like) | LOC100910768 rCG_50143 | 34 | 42 | yes | 2 | 117.16 | Trypsin  group |
| FAGDDAPR | 8 | 847.38243 | A0A8I6AQR0 | Actin, cytoplasmic 2 | Actg1 | 21 | 28 | yes | 2 | 116.54 | Trypsin  group |
| AGFAGDDAPR | 10 | 975.44101 | A0A8I6AQR0 | Actin, cytoplasmic 2 | Actg1 | 19 | 28 | yes | 2 | 109.71 | Trypsin  group |
| PAPPKPEPK | 9 | 959.54402 | A0A0G2K9F6 | Non-histone chromosomal protein HMG-17 | Hmgn2 | 28 | 36 | yes | 2 | 108.46 | Trypsin  group |
| TVRNDITLL | 9 | 1043.5975 | F1MA56 | Chymotrypsinogen B | Ctrb1 | 116 | 124 | yes | 2 | 106.36 | Trypsin  group |
| SEAGGRSEP | 9 | 888.39372 | A0A0G2KA68 | Zinc finger protein 646 | Zfp646 | 1099 | 1107 | yes | 2 | 103.91 | Trypsin  group |
| VVNEDPNAR | 9 | 1012.4938 | Q70AM4 | Kinesin 13B | Kif13b kif13B | 358 | 366 | yes | 2 | 102.72 | Trypsin  group |
| SQSEIGDASR | 10 | 1048.4785 | A0A8I5Y6H8 | Filamin A | Flna | 2032 | 2041 | yes | 2 | 100.25 | Trypsin  group |
| GFAGDDAPR | 9 | 904.40389 | A0A8I6AQR0 | Actin, cytoplasmic 2 | Actg1 | 20 | 28 | yes | 2 | 97.798 | Trypsin  group |
| LGEHNINVLEGDEQFINAAK | 20 | 2210.0968 | P00762 | Serine protease 1, EC 3.4.21.4 (Anionic trypsin I) (Anionic trypsin-1) (Beta-trypsin) (Cationic trypsinogen) (Pretrypsinogen I) (Trypsin I) (Trypsin-1) | Prss1 Trp1 Try1 Tryp1 | 73 | 92 | yes | 2; 3 | 49.423 | Trypsin  group |
| DHFNNLPVNISLSDVQVPTN | 20 | 2222.0968 | A0A8I5ZZ76 | Voltage-dependent calcium channel subunit alpha-2/delta-3 | Cacna2d3 | 64 | 83 | yes | 3 | 8.1875 | Trypsin  group |
| KMVSCDDPLLEEGCRASCFC | 20 | 2204.9033 | Q9WUY0 | Acidic epididymal glycoprotein D/E | Crisp-1 | 25 | 44 | yes | 2 | 4.8909 | Trypsin  group |
| EWLKVPCLSTRLINPENMGF | 20 | 2346.1864 | A0A8I6B374 | Sacsin molecular chaperone | Sacs | 1430 | 1449 | yes | 3 | 4.1864 | Trypsin  group |
| TQDEEDELSSASEESVLSVP | 20 | 2149.9386 | D3ZMG1 | GON-4-like protein | Gon4l | 1472 | 1491 | yes | 3 | 4.1063 | Trypsin  group |
| SLPLQEDFVYHWKAITHYYI | 20 | 2522.2634 | D4A3I5 | FHF complex subunit HOOK-interacting protein 2A (LOC361774 (Predicted)) | Fhip2a Fam160b1 RGD1306116_predicted rCG_57805 | 19 | 38 | yes | 3 | 4.028 | Trypsin  group |
| FRREQELRREREEEQRRRQE | 20 | 2814.4458 | A0A0G2K862 | Trichohyalin | Tchh | 81 | 100 | yes | 3 | 3.1438 | Trypsin  group |
| QQHMKFGKKCWRKVWALLYA | 20 | 2520.3399 | B2RYG7 | Docking protein 3 (Downstream of tyrosine kinase 3) | Dok3 | 15 | 34 | yes | 3 | 3.1438 | Trypsin  group |
| VCQSMGWFPEPRTEWRDPTG | 20 | 2378.0572 | A0A8I5ZZC1 | Erythroblast membrane associated protein (Scianna blood group) | Ermap | 188 | 207 | yes | 3 | 3.1438 | Trypsin  group |
| KLYKCSECDKCFTDEYRLRN | 20 | 2513.1501 | A0A8I5ZWD1 | Uncharacterized LOC102547235 | LOC102547235 | 305 | 324 | yes | 3 | 3.0979 | Trypsin  group |
| SSGCPPLPPRRGAPAGWLSH | 20 | 2042.0268 | A0A0H2UI28 | Brain-derived neurotrophic factor, BDNF | Bdnf rCG_26305 | 11 | 30 | yes | 3 | 3.0979 | Trypsin  group |
| VCMRVCRTWSRWCYDKRLWP | 20 | 2643.2596 | D4A2L9 | F-box and leucine-rich repeat protein 19 (F-box and leucine-rich repeat protein 19 (Predicted), isoform CRA_a) | Fbxl19 Fbxl19_predicted rCG_39403 | 307 | 326 | yes | 3 | 3.0979 | Trypsin  group |
| GRRKWYAYEQYGMYRCLFCS | 20 | 2579.1661 | D3ZYC1 | Zinc finger protein 507 (Zinc finger protein 507 (Predicted), isoform CRA_a) | Zfp507 Znf507_predicted rCG_53890 | 219 | 238 | yes | 3 | 3.0529 | Trypsin  group |
| QALVAIFLCILTITVITLLI | 20 | 2170.3415 | F1M7E5 | Cadherin-5 (Vascular endothelial cadherin) | Cdh5 | 542 | 561 | yes | 2 | 1.5793 | Trypsin  group |
| LTDGFQKKFEQHLRKRFILQ | 20 | 2531.4125 | D3ZM38 | SPATA31 subfamily D member 1 (Uncharacterized protein RGD1565972_predicted) | Spata31d1 Fam75d1d RGD1565972_predicted Spata31d1d rCG_24260 | 579 | 598 | yes | 2 | 0.63122 | Trypsin  group |
| HAVSEGTK | 8 | 827.41373 | M0RBQ5 | Histone H2B | H2bu1 rCG_32940 | 110 | 117 | yes | 2 | 151.56 | Trypsin  group |
| TVLDSGISEVR | 11 | 1174.6194 | A0JN13 | Alpha-crystallin A chain | Cryaa rCG_60707 | 55 | 65 | yes | 2 | 144.09 | Trypsin  group |
